# Incidence of tongue carcinoma in Trinidad and Tobago, West Indies

**DOI:** 10.3892/ol.2015.2862

**Published:** 2015-01-12

**Authors:** MICHAEL J. RAMDASS, AVIND HARRACKSINGH, KHEMANAND MAHARAJ, QUILLAN YOUNG SING, JUSTIN MOOTEERAM, SHAHEEBA BARROW

**Affiliations:** 1 Department of Surgery, University of the West Indies, Port of Spain General Hospital, Port-of-Spain; 2 Department of Dental Surgery, Mount Hope Hospital, Mount Hope, Republic of Trinidad and Tobago

**Keywords:** tongue carcinoma, West Indies

## Abstract

The incidence of tongue carcinoma in Trinidad and Tobago and the greater West Indies is unknown; therefore, the present study examines the frequency of tongue carcinoma cases, drawing comparisons to worldwide and regional data. A retrospective analysis of all confirmed cases of tongue carcinoma was conducted using eight years of data from the pathology records at the Port of Spain General Hospital (Port of Spain, Trinidad and Tobago). A total of 26 cases were confirmed, of which 21 were male (81%) and five were female (19%). The age range was 29–86 years, with a mean age of 57 years, and the most common group affected was the 61–70 years age group. In addition, the number of newly diagnosed cases per year ranged between one and seven, with an average of 3.25 new cases per year and a peak incidence of seven new cases in the year of 2009. In the 19 cases where the degree of differentiation was recorded, histological analysis revealed the extent of differentiation as follows: Five cases (26%) were poorly-differentiated squamous cell carcinoma (SCC); eight cases (42%) were moderately-differentiated SCC; and six cases (32%) were well-differentiated SCC. In addition, one case of chronic inflammatory process and one case of mucoepidermoid adenocarcinoma of the tongue in a 57-year-old female were identified. Overall, the incidence of tongue carcinoma in Trinidad and Tobago appears to be low, estimated at 0.46/100,000 individuals/year. The male:female ratio is 4:1 and SCC is the dominant cancer type (96% of cases). The peak age of occurrence is at 61–70 years. These findings are in agreement with previously determined global data, however, additional research of the risk factors and outcomes of surgery as a treatment strategy for tongue carcinoma is required.

## Introduction

The estimated global annual incidence of oral cancer is 275,000 cases, with a higher incidence in developing countries and >90% classified as squamous cell carcinoma (SCC) (1). Clinical presentation includes a persistent red or white patch, a sore throat or a chronic ulcer. There may also be pain upon swallowing and unexplained otalgia. Associated causes include tobacco use and the human papilloma virus. The basis of treatment includes biopsy to confirm the diagnosis, followed by surgery, radiotherapy and chemotherapy. Lymph node neck dissection is necessary in all cases. Tongue SCC is the most prevalent type of oral cancer, accounting for 25–40% of oral SCCs (2). The 5-year survival rate of tongue cancer is 78% for local spread, 63% for regional spread and 36% for distant spread according to the American Cancer Society (3). Trinidad and Tobago are the southern-most islands in the Caribbean sea, adjacent to the coast of Venezuela, and are composed of a diverse mix of ethnicities, including individuals of Indian descent (40%), African descent (38%), mixed ethnicity (20%) and individuals of European, Chinese and Arabic descent (2%). At present, no epidemiological data exists for tongue SCC in Trinidad and Tobago and the West Indies. Therefore, the present study was conducted to provide data on this rare but preventable malignancy.

## Patients and methods

### Data collection

Retrospective data regarding all cases of tongue histology were collected from the electronic records of the Department of Pathology at the Port of Spain General Hospital (POSGH; Port of Spain, Trinidad and Tobago) from the period between October 2003 and February 2012. Data included information regarding the date of collection, patient age and gender, department and hospital of origin, details of the morphological appearance of the specimen, and histological diagnosis. The data included patients from the following hospitals: The POSGH, the Sangre Grande District General Hospital (SGH) and the Scarborough Regional Hospital (SRH), which are the main referral centres in Trinidad and Tobago, serving a catchment area of ~700,000 individuals and managing the majority of complex tumour cases.

### Statistical analysis

Demographic and epidemiological tongue carcinoma data were analysed using IBM SPSS software version 20 (SPSS, Inc., Armonk, NY, USA) and comparisons were made with previously published worldwide and regional data. Ethical approval was granted from the North West Regional Health Authority (Port of Spain, Trinidad) to collect and analyse the data.

## Results

**Figure 1:**
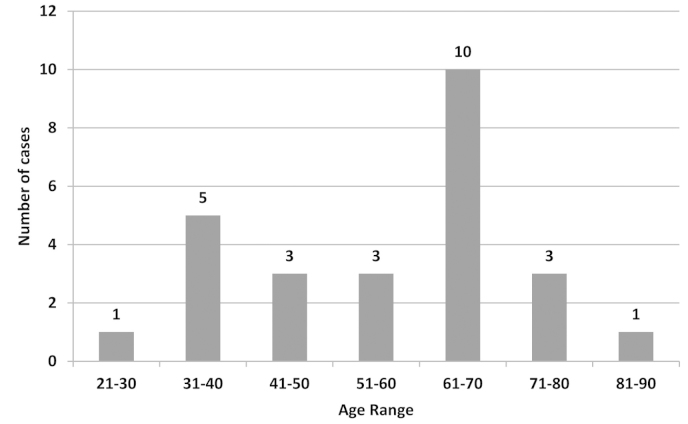
Chart demonstrating the age distribution of tongue carcinoma cases in Trinidad and Tobago.

**Figure 2:**
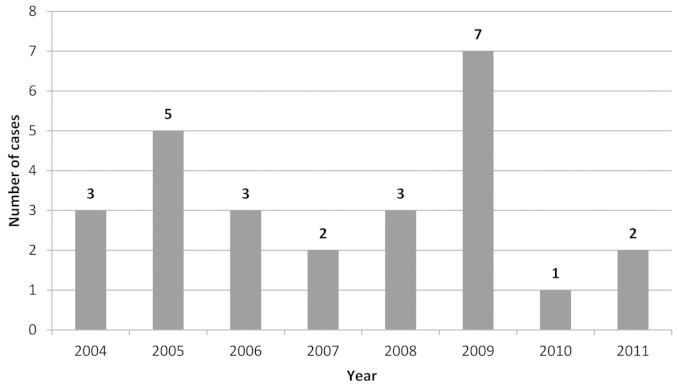
Chart demonstrating the number of new cases of tongue carcinoma occurring annually in Trinidad and Tobago.

Between the study period of October 2003 and February 2012 (101 months), 118 tongue lesions were biopsied. Of these, 26 cases of tongue carcinoma were identified, including 21 cases in males (81%) and five in females (19%). The age range was 29–86 years, with a mean, median and mode of 57, 62 and 66 years, respectively. Furthermore, the most common group affected was the 61–70 years age group (Fig. 1). The number of newly diagnosed cases per year ranged between one and seven, with an average of 3.25 new cases/year and a peak of seven new cases in 2009 (Fig. 2). Analysis of ~700,000 individuals over an eight-year period was used to calculate the incidence rate of tongue carcinoma in Trinidad and Tobago as 0.46 per 100,000 individuals/year. In terms of the hospital of origin, 25 cases were from POSGH (96%) and one case was from SRH (4%); no cases occurred at SGH.

Histological analysis of the 19 cases in which the degree of differentiation was recorded, revealed that the extent of differentiation was distributed as follows: Five cases (26%) of poorly-differentiated SCC, eight cases (42%) of moderately-differentiated SCC and six cases (32%) of well-differentiated SCC. The extent of differentiation was not documented in seven of the cases. In addition, there was one case in which the patient presented with a chronic inflammatory process. There was also one case of mucoepidermoid adenocarcinoma of the tongue in a 57-year-old female originating from POSGH.

## Discussion

Oral cancer is the sixth most common type of carcinoma globally, with an estimated annual incidence of 275,000. It is known to occur more frequently in developing countries and >90% of carcinoma cases in developing countries are classified as SCC (1). It is estimated that carcinoma of the tongue accounts for 25–40% of all SCC lesions (2) and occurs more frequently in developing countries (1), with wide geographical variation. There is a documented high incidence of tongue carcinoma in South and Southeast Asia (Sri Lanka, India, Pakistan and Taiwan), parts of Western Europe (France), Eastern Europe (Hungary, Slovakia and Slovenia), parts of Latin America and the Caribbean (Brazil, Uruguay and Puerto Rico) and Pacific regions (Papua New Guinea and Melanesia) (1). Furthermore, male incidence rates of tongue carcinoma in India have been reported at <6.5 per 100,000 individuals per annum, with corresponding rates in France of <8.0 per 100,000 individuals per annum (4). It is important to analyze the differences in order to make comparisons between developed and developing countries as well as to establish differences and trends between developing countries in order to determine if other environmental and economic factors affect the incidence of tongue cancer.

A recent study performed by Effiom *et al* (5) in Lagos, Nigeria, demonstrated that oral SCC accounted for 10.8% of all tumours biopsied in a cohort with a mean age of 45 years and a male:female ratio of 1.4:1. In total, 40% of these patients were <40 years old and the peak incidence was in the 20–29 and 40–49 years age groups. Poorly-differentiated SCC was the most common subtype of oral SCC identified (47.6%), followed by well-differentiated (32.6%) and moderately-differentiated (19.7%) SCC. This is in contrast to the Trinidadian data of the present study, where the male:female ratio was closer to 4:1, the peak incidence was in the 61–70 years age group and moderately-differentiated SSC was the most common type of oral SCC, followed by well-differentiated and then poorly-differentiated SCC.

A retrospective study examining the treatment of tongue cancer between 1996 and 2002 in Mumbai, India (6) identified the most common age range of presentation as 50–60 years and the male:female ratio as 4:1, corresponding to the distribution in Trinidad and Tobago. The majority of cases were oral SCC (98%), with a small number of mucoepidermoid (1%) and adenocystic carcinoma (1%) cases additionally reported. Of the oral SCC, well-differentiated SCC (77.8%) was the most frequent subtype identified, followed by moderately differentiated (20%) and poorly differentiated (2.2%) SCC; this is in contrast to the present study, in which moderately distributed SCC was the most prevalent type, followed by well-differentiated and poorly-differentiated SCC.

A study of patients presenting to Mexico City’s General Hospital (Mexico City, Mexico) with oral SCC between 1990 and 2008 (7) identified that 58.4% of patients were male, as opposed to the 81% male predominance identified in the present study, thus resulting in a male:female ratio of 1.4:1. In addition, the mean age was 62.5±14.9 years, which was higher than the mean age of 57 years that was calculated for the present study. The tongue (44.7%) was the most common anatomical region of oral SCC, followed by the lips (21.2%) and gums (20.5%), while the most frequent histological degree was moderately-differentiated SCC (61.2%).

A retrospective analysis of patients with oral SCC of the tongue in Lisbon, Portugal, for the period of 2001–2009 (8) identified that 71% of patients were male, with a male:female ratio of 2.45:1. The mean age of occurrence in the male patients was within the fifth decade (58.8 years), corresponding to the mean age of 57 years identified in the present study, and within the sixth decade (65) for females. In addition, the peak incidence of tongue carcinoma was within the 61–70 years age group for males and females, similar to the present study.

A recent study of SSC of the oral tongue (OTSCC), the base of the tongue and the tonsils in the United States (9) identified that 58.6% of OTSCC cases occurred in males and 41.4% occurred in females, with a male:female ratio of 1.42:1. The peak incidence of OTSCC was 60–79 years (45.2%), in agreement with the peak incidence age group of 61–70 years identified in the present study, followed by 40–59 years (36.3%).

In conclusion, the incidence of tongue carcinoma in Trinidad and Tobago is low in comparison to other regions, which may be due to the fact that tobacco chewing is rare in this region, and the incidence has with a male to female ratio of 4:1 with 96% of cases classified as SCC. In the present study, the peak incidence was found in individuals aged 61–70 years, with an overall incidence of 0.46 per 100,000 individuals per annum. These findings are in agreement with certain previously reported global data, with no detectable differences identified between ethnic variations in Trinidad and Tobago. Additional studies are required to determine if the differences identified are due to the limitations of the sample size, and closer analysis is required to identify the risk factors and outcomes of surgery for the treatment of tongue carcinoma in Trinidad and Tobago. The present study adds previously unreported data to the regional and world dataset and may serve as a platform for additional research on the topic.
